# Cross-Sectional Analysis of the Correlation Between Daily Nutrient Intake Assessed by 7-Day Food Records and Biomarkers of Dietary Intake Among Participants of the NU-AGE Study

**DOI:** 10.3389/fphys.2018.01359

**Published:** 2018-10-01

**Authors:** Rita Ostan, Giulia Guidarelli, Enrico Giampieri, Catia Lanzarini, Agnes A. M. Berendsen, Olga Januszko, Amy Jennings, Noëlle Lyon, Elodie Caumon, Rachel Gillings, Ewa Sicinska, Nathalie Meunier, Edith J. M. Feskens, Barbara Pietruszka, Lisette C. P. G. M. de Groot, Susan Fairweather-Tait, Miriam Capri, Claudio Franceschi, Aurelia Santoro

**Affiliations:** ^1^Department of Experimental, Diagnostic and Specialty Medicine, Alma Mater Studiorum, University of Bologna, Bologna, Italy; ^2^Interdepartmental Centre “L. Galvani” (CIG), Alma Mater Studiorum, University of Bologna, Bologna, Italy; ^3^Department of Physics and Astronomy, Alma Mater Studiorum, University of Bologna, Bologna, Italy; ^4^Department of Human Nutrition, Wageningen University, Wageningen, Netherlands; ^5^Department of Human Nutrition, Warsaw University of Life Sciences-SGGW, Warsaw, Poland; ^6^Norwich Medical School, University of East Anglia, Norwich, United Kingdom; ^7^Centre Hospitalier Universitaire de Clermont-Ferrand, Clermont-Ferrand, France; ^8^Institute of Neurological Sciences (IRCCS), Bologna, Italy

**Keywords:** NU-AGE, 7-day food records, nutrient intake, Mediterranean diet, aging

## Abstract

Methods for measuring diet composition and quantifying nutrient intake with sufficient validity are essential to study the association between nutrition and health outcomes and risk of diseases. 7-day food records provides a quantification of food actually and currently consumed and is interesting for its use in intervention studies to monitor diet in a short-term period and to guide participants toward changing their intakes. The objective of this study is to analyze the correlation/association between the daily intake of selected nutrients (collected by a 7-day food records plus a mineral/vitamin supplementation questionnaire) and estimates of energy expenditure as well as blood and urine biomarkers of dietary intakes in 1,140 healthy elderly subjects (65–79 years) at baseline of the NU-AGE intervention study (NCT01754012, clinicaltrials.gov). The results show that: the daily intake of energy correlated significantly with predicted total energy expenditure (pTEE) (ρ = 0.459, *p* < 0.001, and *q* < 0.001); protein intake correlated significantly with the ratio of 24 h urinary urea to creatinine excretion (ρ = 0.143 for total protein intake, ρ = 0.296 for animal protein intake, and ρ = 0.359 for protein intake/body weight, *p* < 0.001 and *q* < 0.001 for each correlation); vitamin B12 and folate intakes correlated significantly with their serum concentrations (ρ = 0.151 and ρ = 0.363, respectively; *p* < 0.001 and *q* < 0.001 for each correlation); sodium and potassium intakes correlated significantly with their 24 h urinary excretion (ρ = 0.298 and ρ = 0.123, respectively; *p* < 0.001 and *q* < 0.001 for each correlation); vitamin B12 and folate intakes were negatively associated with plasma homocysteine measure (*p* = 0.001 and *p* = 0.004, respectively); stratifying subjects by gender, the correlations between energy intake and pTEE and between potassium intake and its 24 h urinary excretion lost their significance in women. Even if the plasma and urinary levels of these nutrients depend on several factors, the significant correlations between daily reported intake of nutrients (protein, vitamin B12, folate, and sodium) and their blood/urinary markers confirmed that the 7-day food records (plus a supplementation questionnaire) provides reliable data to evaluate short-term current dietary intake in European elderly subjects and it can be exploited to guide and monitor NU-AGE participants through the shift of their diet according NU-AGE recommendations.

## Introduction

The worldwide population is progressively aging, with future economic and society consequences. While life expectancy continues to rise, health span is not keeping pace. Aging is the most important risk factor for the majority of chronic diseases and current therapies often decrease mortality without preventing or reversing the decline in overall health (Kennedy et al., 2014). The urgent need to extend health span suggests the importance to identify the potential interactive association between nutrition, lifestyle, genetics, and the risk of chronic disease. Therefore, modern epidemiology is aspiring to consider assessment of dietary intakes along with other fundamental variables such as lifestyle/environmental risk factors and genetic predisposition. However, the individual dietary exposure is characterized by an high heterogeneity and a multi-factorial complexity and all the methods aimed to assess dietary intakes are associated with a proportion of measurement error (Giampieri et al., submitted) that may distort/attenuate the association between nutrient intake and/or nutritional status and disease risk or the effects of a dietary intervention. The assessment of the quantitative and qualitative characteristics of the whole diet, rather than the study of single nutrients, is becoming increasingly urgent (Russell et al., 2017). Most of the epidemiological studies evaluating the adherence to the Mediterranean diet and its association with health outcomes converged to utilize different versions of food frequency questionnaires to rapid estimate the long-term usual intakes with a low burden for the participant (Willett, 2013). This approach is valid and suitable for a qualitative evaluation of dietary habits but, the use of 7-day food records, taking into account portion sizes, is a validate tool to provide a quantification of food actually and currently consumed (Willett, 2013; Bingham et al., 2017) well estimating the average short-term nutrient intake (Willett, 2013; Ortega et al., 2015; Yuan et al., 2017). Therefore, this method is interesting for its use in epidemiological studies to monitor diet in a brief period and to guide participants in intervention studies toward changing their intakes. However, collecting data about dietary intake involves many challenges due to their complexities and limitations (Kaaks and Riboli, 2005).

Dietary biomarkers as objective and independent measures may be used to verify the accuracy of dietary intakes assessment methods in dietary intervention trials and observational risk association studies. Existing biomarkers of nutrient intakes are not “ideal,” but they are functional and have found wide spread applicability in modern nutritional epidemiology (Jenab et al., 2009; Shim et al., 2014). Biomarkers of exposure, such as urinary sodium and potassium as well as urinary biomarkers of protein consumption, generally have a short time course. For these biomarkers, an accurate short-term measure of intake, i.e., food records, should have a good correlation if the dietary intake method is accurate. For others biomarkers (blood concentration of vitamins), there may be quite a longer time before a steady state is achieved, and food frequency questionnaires may be more appropriate. However, also these long-term biomarkers can be evaluated by 7-day food records with the caveat that the dietary intake measurement reflects longer-term intake and could be considered representative of the habits of the subject. Nevertheless, the information about the real correlation between dietary intakes (assessed by 7-day food records) and blood/urine biomarkers of nutrient intakes are still limited and obtained on a restricted number of subjects (Day et al., 2001; McKeown et al., 2001).

The Mediterranean Diet is a widely studied healthy dietary pattern, providing an equilibrated mix of nutrients with antioxidant, anti-inflammatory, and prebiotic activity, able to elicit an integrated network of cellular mechanisms of stress/oxidative damage response (Martucci et al., 2017). The key role of Mediterranean Diet for the prevention of a wide range of chronic age-related pathologies has been extensively demonstrated (Talegawkar et al., 2012; Sofi et al., 2013; Johnston, 2014; Trichopoulou et al., 2014; Ostan et al., 2015; Valls-Pedret et al., 2015). NU-AGE project was aimed to investigate the effect of a newly designed, personally tailored Mediterranean dietary pattern, designed to meet the dietary recommendations of people over 65 years of age (NU-AGE diet plus vitamin D supplementation) on inflammaging process (i.e., the chronic low grade pro- inflammatory status typical of aging representing the common biological denominator of main age-related chronic diseases and geriatric syndromes) (Franceschi et al., 2000; Monti et al., 2017) and the consequent decline of function at the level of different organs and systems in elderly (Berendsen et al., 2014).

No study so far applied a validate instrument such as the 7-day food record in unprecedented consistent elderly well characterized European population with the characteristics of the subjects recruited in the NU-AGE study (1,140 healthy European men and women aged 65–79 years from 5 European countries). Considering that the analysis of the 7-day food records is pivotal for all the subsequent studies aimed to evaluate the effect of the NU-AGE diet, the objective of the present work is to verify if the data obtained by the dietary assessment method chosen for the NU-AGE dietary intervention are coherent and reliable. Thus, the correlation/association between the daily intake of selected nutrients (resulting from a 7-day food records plus a mineral/vitamin supplementation questionnaire), and blood and urine biomarkers of their intakes at baseline of the NU-AGE dietary intervention was analyzed. The selection of nutrients has been driven by their importance for the health of elderly people. In particular, the daily protein intake is a fundamental nutritional issue for the maintenance of muscle protein synthesis, muscle mass and function and nutritional interventions to optimize protein intake patterns (completed by physical exercise protocol) have emerged as important approaches to manage sarcopenia in aging (Deer and Volpi, 2015). Elderly people are at increased risk of B vitamin deficiencies due to decreased food intake and increased malabsorption. Low folate and vitamin B12 status and increase in homocysteine concentration have been associated with different adverse health outcomes (cognitive impairment, stroke, fractures, and cancer) (Mendonça et al., 2016c). Dietary sodium and potassium intake have been found to be related to hypertension, and moderate dietary salt reduction together with an increase in potassium intake causes significant reductions in blood pressure reducing the risk of cardiovascular disease (Binia et al., 2015; Okayama et al., 2016).

Thus, in the present work, the daily intake of energy will be correlated with the predicted total energy expenditure (pTEE), protein intake with urine markers of protein consumption (the ratio of 24 h urinary urea to creatinine excretion), vitamin B12 and folate intakes with their serum levels, sodium and potassium intakes with their urinary measures and vitamin B12, B2, B6, and folate intake with plasma homocysteine measure in 1140 NU-AGE healthy elderly participants (65–79 years) taking also into account gender and country differences.

## Materials and methods

### Study design

This study was performed using baseline data of the NU-AGE dietary intervention study. The NU-AGE study is a 1-year, randomized, parallel trial to investigate whether a newly designed, personally tailored Mediterranean dietary pattern diet, designed to target dietary recommendations for people over 65 years of age (NU-AGE diet plus vitamin D supplementation) can counteract or slow down the inflammaging process. The study was carried out in five European study centers (Bologna in Italy, Norwich in the UK, Wageningen in The Netherlands, Warsaw in Poland, and Clermont-Ferrand in France). Recruitment started in April 2012 and finished in January 2014 including 1,296 healthy European men and women aged 65–79 years. Study participants were free living and responsible for their own dietary choices. Exclusion criteria included any clinically diagnosed chronic disease, use of corticosteroids or insulin medications, recent use of antibiotics or vaccinations, recent change in habitual medication, presence of food allergy or intolerance necessitating a special diet, presence of frailty according to the Fried criteria (Fried et al., 2001) or malnutrition [defined as BMI (kg/m^2^) <18.5 or >10% weight loss in the previous 6 months).

The rationale and design of this intervention study are described in detail elsewhere (Berendsen et al., 2014; Santoro et al., 2014). Briefly, participants completed questionnaires about their health and lifestyle and a 7-day food record (accomplished by a mineral/vitamin supplementation questionnaire). Local ethical approval was provided by the Independent Ethics Committee of the Sant'Orsola-Malpighi Hospital Bologna (Italy), the National Research Ethics Committee — East of England (UK), the Wageningen University Medical Ethics Committee (Netherlands), the Bioethics Committee of the Polish National Food and Nutrition Institute (Poland) and South-East 6 Person Protection Committee (France). All study procedures were in accordance with the ethical standards of the Helsinki Declaration. All participants gave informed consent before participating. The trial was registered at clinicaltrials.gov (NCT01754012).

### Dietary assessment

Dietary intake was assessed by means of a validate version of the 7-day food records (Ortega et al., 2015) completed by the participants. To obtain accurate data, participants were trained one to one receiving exhaustive instructions before starting to fill in the 7-day food records. A trained interviewer explained to participants how to record detailed information about food preparation methods, ingredients of mixed dishes and recipes, and, if possible, the brand name of commercial products. 7-day food records were provided in a structured format, with tables for each day and eating occasion (before breakfast, breakfast, morning snacks, lunch, afternoon snacks, dinner, evening snacks, night snacks), time/hour, location, foods, and drinks consumed, quantity and recipes in order to record all details of the meals (see Supplementary Material for a structured format of the 7-day food records employed in NU-AGE). The amounts of each food could be measured with a kitchen weighing scale, using household measures (bowls, cups, glasses, spoons, etc.) and two-dimensional aids such as photographs (showing three portion sizes options for each example items) provided to each participant during the dietary recording period. Participants were recommended to record data at the time the foods were eaten/consumed and not to change eating habits during the week of registration. Seven consecutive days of food record minimizes the bias related to the difference in meal consumption due to a particular day of the week (i.e., during the weekend) and allows to include information about foods eaten occasionally (Shim et al., 2014). At the end of the recorded period, during an in-depth interview with a trained dietician/research nutritionist, the 7-day food records were accurately checked to obtain more detailed information about types of foods, dressings, preparation methods and recipes, to estimate portion sizes by using real-life models and pictures and to probe the possible consumption of forgotten foods. Consumed foods were coded according to standardized coding procedures and translated into nutrients by the use of software exploiting local food composition tables (NEVO 2011 in The Netherlands, WISP in The UK, INRAN and IEO in Italy, NFNI in Poland and CIQUAL French food composition table in France). In order to estimate the variability of the food composition databases among NU-AGE study centers, 16 very basic foods (semi-skimmed milk, egg, apple, orange, chicken breast, beef filet, cod filet, salmon, tomatoes, peas, nuts, potatoes, lager beer, read brown whole meal, spinach, extra-virgin olive oil) were analyzed in each NU-AGE study center for their calories and nutrient content per 100 grams. For energy content and each nutrient, the median absolute deviation was estimated among countries for each food. This was standardized to the mean value of the energy and nutrient content to obtain a percentage of variation per food among centers. Then, the median of this percentage of variation over all the foods has been estimated to obtain a per-nutrient percentage variability among NU-AGE study centers (Supplementary Table 2).

Energy (kcal), total protein (g), animal protein (g), protein intake normalized on body weight (protein intake/BW, g/kg BW), alcohol (g), and sodium intake (g) calculated by the 7-day food records were used in the analysis. The daily total intakes of vitamin B2 (mg), vitamin B6 (mg), vitamin B12 (μg), folate (μg), and potassium (mg) for the analysis were obtained adding the dietary intakes calculated by the 7-day food records to the intakes resulting from mineral/vitamin supplementation (assessed by a specific supplements questionnaire).

### Study population

Participants who had not completed the 7-day food records at baseline, those with missing data on supplement use, incomplete diet data were excluded. Data collected from a total of 1,140 participants (242 from Italy, 248 from the UK, 241 from the Netherlands, 220 from Poland, and 189 from France) were considered for the analysis.

### Laboratory measures

Vitamin B12 and folate were measured on serum by chemioluminescence (ADVIA CENTAUR XP by SIEMENS) and homocysteine was measured on plasma by enzymatic method (Olympus AU400 chemistry analyzer by Beckman) according to clinical standardized methods.

A 24-h urine collection was obtained for estimation of sodium, potassium, urea, and creatinine excretion. Participants were given written and verbal instructions for the 24-h collection. The first urine of the day was discarded and all urine over the following 24 h, including the first urine on the second day, was collected in standard containers that contained 2.7 ml of 1% sodium azide solution. Urinary sodium and potassium were measured by direct potentiometry assay (Italy: Olympus AU400 chemistry analyzer by Beckman), ion selective electrod method (UK: Abbott Architect C 16000; The Netherlands: Roche 917 Anlyzer; Poland Cobas 6000 Analyzer) or indirect potentiometry method (Dimension Vista system of SIEMENS society). Urinary creatinine was measured by clinical standardized methods (Italy, The Netherlands and Poland: colorimetric method based on the Jaffe reaction; UK: enzymatic Trinder method; France: cinetic bichromatic technique) and urinary urea by spectrophotometric enzymatic method using dehydrogenase glutamate urease. Completeness of 24-h urine collections was assessed based on expected creatinine excretion (mg/d) in relation to body weight (kg); individuals with values outside the expected range (14.4–33.6 for men and 10.8–25.2 for women) were excluded (*n* = 169, 68 men, and 101 women). The ratio of 24 h urinary urea to creatinine excretion (urea:creatinine excretion) was considered as a biomarker of protein intake (Tay et al., 2015).

### Other variables

Height was measured by a stadiometer to the nearest 0.1 cm. Weight was measured to the nearest 0.1 kg with a calibrated scale while wearing light clothes. Body Mass Index (BMI) was calculated as weight/height^2^ (kg/m^2^). All measures were taken by trained research assistants. Chewing difficulties and the use of removable dentures were self-reported by specific questions. Appetite was assessed using SNAQ (maximum score 20, score <14 indicates poor appetite) (Wilson et al., 2005). Depression status was investigated by Geriatric Depression Scale short form (GDS, 15 items), a cut off value of 5 was considered as indicative of depression symptoms (Yesavage et al., 1982).

Basal metabolic rate (BMR) was estimated by the Mifflin-St Jeor equation, a revised version of the Harris-Benedict equation (Mifflin et al., 1990).

Possible mis-reporters were defined as having an energy intake:BMR below 1.05 (under-reporters) or over 2.00 (over-reporters) (Mendonça et al., 2016a). Accordingly, 120 under-reporters (10.5% of the participants) and 49 over-reporters (4.3% of the participants) were identified. However, these possible mis-reporters have not been excluded from the analysis because of the uncertainty surrounding this estimate and the small differences observed in the correlation analysis between excluding and not excluding mis-reporters (Mendonça et al., 2016b) (Supplementary Table 2).

Data on physical activity were obtained by the Physical Activity Scale for the Elderly (PASE). Predicted total energy expenditure (pTEE) was calculated as BMR^*^physical activity level (PAL). PAL was considered as 1.2 for sedentary subjects (physical activity frequency <1 time per week), 1.375 for lightly active subjects (physical activity frequency 1–3 times per week), 1.55 for moderately active subjects (physical activity frequency 4–5 times per week), 1.725 for highly active subjects (physical activity frequency 6–7 times per week), and 1.9 for very highly active subjects (physical activity frequency more than 7 times per week with extra heavy workouts) (Lee and Chan, 2016).

### Statistical analyses

Population characteristics are reported as median [Interquartile range, IQR, i.e., the difference between Q1 and Q3]. According the parametric test Shapiro–Wilk, all the continuous variables are not normally distributed and non-parametric tests were applied. Differences between men and women have been evaluated by Mann-Whitney *U* test for continuous variables and logistic regression for categorical variables (chewing difficulties, use of removable dentures and poor appetite). Correlations between dietary intakes calculated by 7-day food records plus supplement questionnaire and pTEE, blood and urine markers were performed by means of a Spearman's rank correlation. Comparison of correlation between men and women and between countries were performed. When comparing correlations, a test of significance is necessary to control for the possibility of an observed difference occurring simply by chance (Diedenhofen and Musch, 2015). Fisher's z-test was performed to detect differences between correlations of independent groups (men vs. women and between countries). The *p*-values of the correlation analysis were adjusted by the Benjamini-Hockberg correction (*q*-values) for multiple testing (Benjamini and Hochberg, 1995). The association between intake of vitamin B12 and its serum level and between intake of vitamin B2, B6, B12, and folate (essential cofactors for the homocysteine pathway) with plasma level of homocysteine were evaluated by a general linear model with backward elimination, adjusted for age, alcohol intake (which may strongly affect the absorption and the metabolism of B vitamins in elderly) (Haller, 1999) and use of proton pump inhibitors (PPI), using a *p* < 0.05 threshold. For the association analysis, the variables were transformed using a logarithmic transform to standardize the variable distributions, to reduce the effect of outliers and to increase the sensitivity on the low end of the scale. Statistical analyses were carried out using SPSS 23.0 for windows (SPSS Inc., Chicago, IL, USA) and Rstudio for Windows (Version “1.0.136”). A two-sided *p* < 0.05 was considered significant.

## Results

Table 1 shows the general characteristics, pTEE, urinary, and blood biomarkers and daily dietary intakes of the 1,140 NU-AGE participants (508 men and 632 women, median age 71 years) at baseline of the NU-AGE dietary intervention. No age difference was observed between men and women (*p* = 0.186). Only 75 subjects (6.6% of the entire NU-AGE population) reported chewing difficulties although a remarkable number of participants (456 subjects, i.e., 40.0% of the entire NU-AGE population) wore removable dentures. No difference were observed in SNAQ score between men and women (*p* = 0.407) and poor appetite (SNAQ score <14) was recorded only in 49 subjects (4.3% of the entire NU-AGE population). Only 74 subjects (6.5% of the entire NU-AGE population) reported depression symptoms with a higher prevalence among women (*p* < 0.001).

**Table 1:** General characteristics, pTEE, urinary and blood biomarkers and daily dietary intakes of the NU-AGE participants.

	**Total (*n* = 1140)**	**Men (*n* = 508)**	**Women (*n* = 632)**	***p***
Age (years)	70.0 [7.0]	71.0 [7.0]	70.0 [7.0]	0.186
Chewing difficulties, n (%)	75 (6.6%)	23 (4.5%)	52 (8.2%)	0.014
Removable dentures, n (%)	456 (40.0%)	202 (39.8%)	254 (40.2%)	0.914
SNAQ score	16 [2]	16 [1]	16 [2]	0.407
Poor appetite (SNAQ score < 14), n (%)	49 (4.3%)	18 (3.5%)	31 (4.9%)	0.265
GDS score	1 [2]	1 [2]	1 [3]	0.023
Depression symptoms (GDS score > 5), n (%)	74 (6.5%)	20 (3.9%)	54 (8.6%)	<0.001
Weight (kg)	72.4 [19.0]	80.0 [16.0]	66.0 [15.3]	<0.001
BMI (kg/m^2^)	26.2 [4.9]	26.5 [4.3]	25.9 [5.4]	0.005
pTEE (kcal)	2007 [624]	2354 [401]	1723 [337]	<0.001
Vitamin B12 (serum) (pg/mL)	357 [161]	335 [145]	373 [174]	<0.001
Folate (serum) (ng/mL)	8.6 [5.5]	7.8 [4.0]	9.7 [6.6]	<0.001
Homocysteine (plasma) (μmol/L)	11.7 [4.9]	12.6 [4.9]	10.9 [4.2]	<0.001
Urea^a^ (urine) (g/24 h)	20.4 [7.7]	23.8 [8.0]	18.2 [6.3]	<0.001
Creatinine^a^ (urine) (g/24 h)	1.2 [0.6]	1.5 [0.4]	0.9 [0.2]	<0.001
Urea:creatinine^a^ excretion	17.2 [5.2]	15.5 [3.5]	18.9 [5.5]	<0.001
Potassium^a^ (urine) (mmol/L)	38.0 [19.0]	43.0 [23.0]	34.0 [14.0]	<0.001
Sodium^a^ (urine) (mmol/L)	70.0 [46.0]	88.0 [62.0]	59.0 [35.0]	<0.001
Energy intake (kcal)	1828 [572]	2093 [570]	1668 [413]	<0.001
Carbohydrate intake (% energy)	44.1 [9.6]	44.1 [10.0]	44.1 [9.1]	0.695
Lipid intake (% energy)	33.8 [7.1]	33.1 [7.2]	34.7 [7.2]	<0.001
Protein intake (% energy)	16.1 [3.2]	15.7 [3.0]	16.5 [3.3]	<0.001
Carbohydrate intake (g)	214.3 [78.9]	249.4 [86.9]	193.5 [62.4]	<0.001
Lipid intake (g)	68.8 [28.9]	77.6 [31.5]	62.4 [24.2]	<0.001
Total Protein intake (g)	74.0 [22.1]	82.2 [23.6]	69.1 [18.1]	<0.001
Animal protein intake (g)^#^	43.8 [19.2]	48.1 [19.9]	41.8 [18.8]	<0.001
Protein intake/BW (g/kg BW)	1.0 [0.3]	1.0 [0.3]	1.0 [0.3]	0.998
Alcohol intake (g)	5.5 [13.6]	9.5 [17.2]	3.4 [9.8]	<0.001
Vitamin B2 intake (mg)	1.7 [0.9]	1.8 [0.8]	1.6 [0.9]	<0.001
Vitamin B6 intake (mg)	1.8 [0.8]	1.9 [0.8]	1.7 [0.8]	<0.001
Vitamin B12 intake (μg)	5.0 [4.8]	5.3 [5.4]	4.7 [4.5]	0.003
Folate intake (μg)	292 [148]	300 [148]	287 [148]	0.043
Potassium intake (mg)	3145 [1002]	3326 [1145]	3020 [929]	<0.001
Sodium intake (mg)	2346 [1198]	2686 [1314]	2115 [925]	<0.001

*Continuous variables are expressed as median [interquartile range, IQR]. Categorical variables are expressed as number (n) and percentage (%). p-values for continuous variables derived through Mann-Whitney U test while p-values for categorical variables derived from a binary logistic regression for differences between men and women*.

a *169 subjects (68 men and 101 women) were excluded because outside of the expected range of creatinine excretion in relation to body weight*.

# *Animal protein intake was not assessed on French participants*.

The carbohydrate, fat, and protein intake (% of energy) are in line with the calculated energy contributions (%) from grams of macronutrients in Mediterranean diet (Davis et al., 2015). The lipid and protein intake (% of energy) is higher in women than in men (*p* < 0.001 for both comparisons).

Weight, BMI, pTEE, plasma concentration of homocysteine as well as urine concentration of urea, creatinine, potassium, and sodium were higher in men than in women (*p* = 0.005 for BMI, *p* < 0.001 for other variables) (Table 1). Serum concentration of vitamin B12 and folate as well as urea:creatinine excretion are higher in women than in men (*p* < 0.001 for each comparison). The daily median dietary intakes of energy, carbohydrate, fats, total and animal protein, alcohol, vitamin B2, B6, B12, folate, potassium, and sodium were higher in men than in women (*p* = 0.003 for vitamin B12, *p* = 0.043 for folate, *p* < 0.001 for other variables) (Table 1). No difference was observed for the protein intake/BW between men and women (*p* = 0.998) (Table 1).

Table 2 showed the Spearman rank correlation between daily dietary intakes, pTEE, and blood and urine biomarkers in the entire NU-AGE population. Energy intake was significantly correlated with pTEE on the entire NU-AGE population at baseline (ρ = 0.459, *p* < 0.001, and *q* < 0.001). Total protein, animal protein and protein intake/BW were significantly correlated with urea:creatinine excretion (ρ = 0.101, ρ = 0.264, ρ = 0.352, respectively; *p* < 0.001 and *q* < 0.001 for each correlation). The daily intake of vitamin B12 was significantly correlated with its serum level (ρ = 0.151, *p* < 0.001 and *q* < 0.001) and the daily intake of folate was significantly correlated with its serum level (ρ = 0.363, *p* < 0.001, and *q* < 0.001). Finally, the daily intake sodium was significantly correlated with its urinary level (ρ = 0.297, *p* < 0.001, and *q* < 0.001). The correlation between daily intake of potassium and its urinary levels was weak in the entire NU-AGE population (ρ = 0.148, *p* < 0.001, and *q* < 0.001) and no longer significant stratifying subjects by gender (Table 3).

**Table 2:** Spearman rank correlation between daily dietary intakes, pTEE, blood and urine biomarkers in the entire NU-AGE population.

	**ρ (rho)**	**p**	**q**
Energy intake*pTEE	0.459	<0.001	<0.001
Total protein intake*Urea:creatinine excretion^a^	0.101	<0.001	<0.001
Animal protein intake*Urea:creatinine excretion^#^^a^	0.264	<0.001	<0.001
Protein intake/BW (g/kg BW)*Urea:creatinine excretion^a^	0.352	<0.001	<0.001
Vitamin B12 intake*Vitamin B12 (serum)	0.151	<0.001	<0.001
Folate intake*Folate (serum)	0.363	<0.001	<0.001
Potassium intake*Potassium (urine)^a^	0.148	<0.001	<0.001
Sodium intake*Sodium (urine)^a^	0.297	<0.001	<0.001

*ρ (rho) is the Spearman rank correlation coefficient and p the significance of Spearman rank correlation. q-values derive from Benjamini-Hockberg correction of p-values for multiple testing*.

a *169 subjects were excluded because outside of the expected range of creatinine excretion in relation to body weight*.

# *Animal protein intake was not assessed on French participants*.

**Table 3:** Spearman rank correlation between daily dietary intakes, pTEE, blood, and urine biomarkers in men and women enrolled in NU-AGE project.

**Correlation**	**Men**			**Women**			**Men vs. women**	
	**ρ (rho)**	***p***	**q**	**ρ (rho)**	***p***	**q**	***p***	**q**
Energy intake*pTEE	0.206	<0.001	<0.001	0.066	0.099	0.998	0.017	0.319
Total protein intake*Urea:creatinine excretion^a^	0.413	<0.001	<0.001	0.354	<0.001	<0.001	0.284	0.998
Animal protein intake*Urea:creatinine excretion^#^^a^	0.412	<0.001	<0.001	0.401	<0.001	<0.001	0.838	0.998
Protein intake/BW (g/kg BW)*Urea:creatinine excretion^a^	0.423	<0.001	<0.001	0.403	<0.001	<0.001	0.709	0.998
Vitamin B12 intake*Vitamin B12 (serum)	0.234	<0.001	<0.001	0.111	<0.001	<0.001	0.034	0.998
Folate intake*Folate (serum)	0.325	<0.001	<0.001	0.447	<0.001	<0.001	0.016	0.512
Potassium intake*Potassium (urine)^a^	0.065	0.176	0.998	0.062	0.154	<0.998	0.963	0.998
Sodium intake*Sodium (urine)^a^	0.183	<0.001	<0.001	0.185	<0.001	<0.001	0.974	0.998

*Fisher's z-test was performed to detect differences between correlations in men and women. ρ (rho) is the Spearman rank correlation coefficient and p the significance of Spearman rank correlation. Fisher's z-test was performed to detect differences between correlations of men and women. q-values derive from Benjamini-Hockberg correction of p-values for multiple testing*.

a *169 subjects (68 men and 101 women) were excluded because outside of the expected range of creatinine excretion in relation to body weight*.

# *Animal protein intake was not assessed on French participants*.

**Figure 1:**
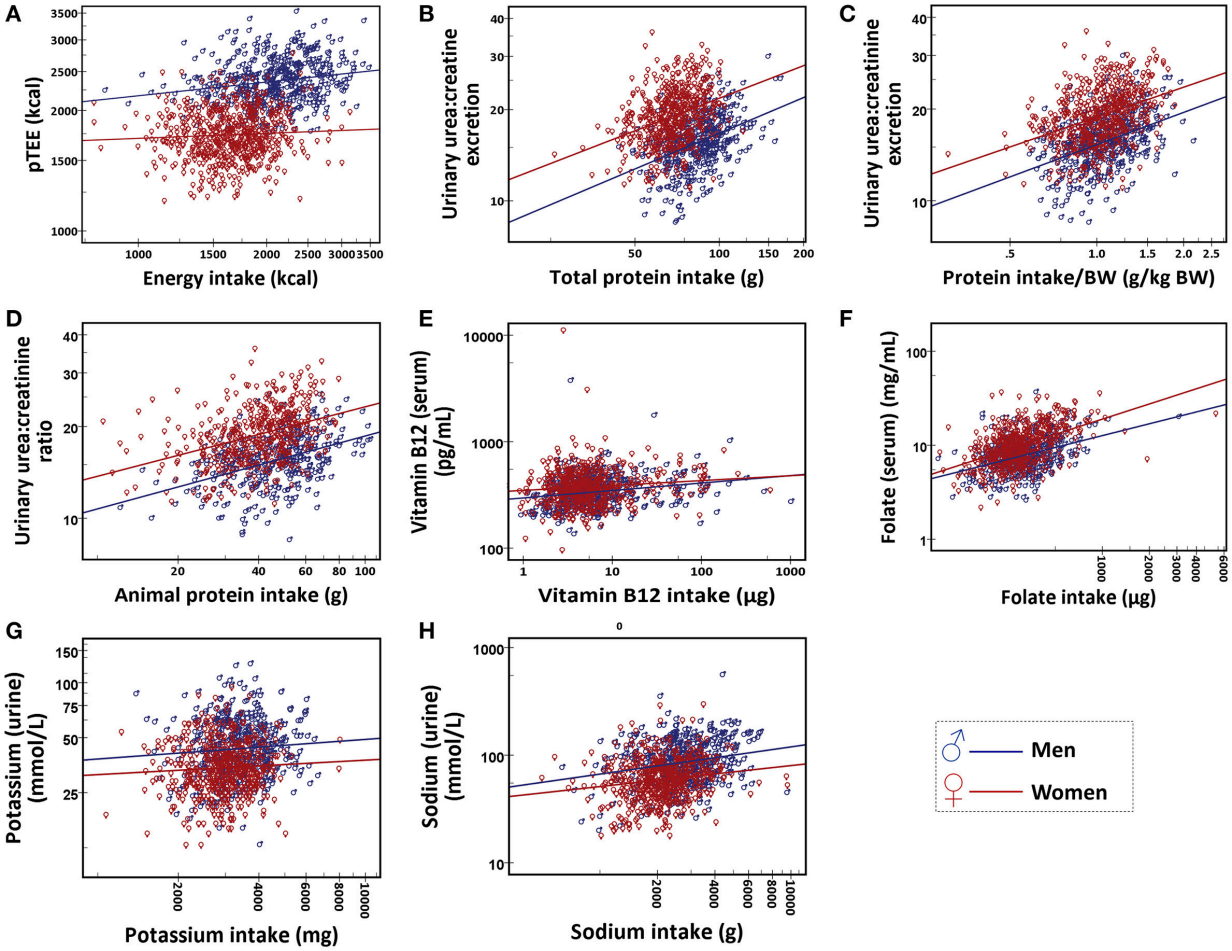
Scatter plot of **(A)** energy intake and pTEE, **(B)**, total protein intake and urea:creatinine excretion, **(C)** protein intake/BW (g/kg BW) and urea:creatinine excretion, **(D)** animal protein intake and urea:creatinine excretion, **(E)** vitamin B12 intake and serum level of vitamin B12, **(F)** folate intake and serum level of folate, **(G)** potassium intake and urinary potassium and **(H)** sodium intake and urinary sodium. Data are shown for men (in blue) and women (in red).

Table 3 showed the Spearman rank correlation between daily intakes, pTEE, and blood and urine biomarkers in men and in women and the last two columns showed the comparison of the correlation between men and women. Figure 1 showed the scatter plots of the distribution of each pair of variables. Stratifying subjects by gender, energy intake was significantly correlated with pTEE only in men (ρ = 0.206, *p* < 0.001, and *q* < 0.001). Total and animal protein intake as well as protein intake/BW were significantly correlated with urea:creatinine excretion both in men and in women (ρ = 0.413, ρ = 0.412, and ρ = 0.423 respectively in men, ρ = 0.354, ρ = 0.401, and ρ = 0.403 respectively in women, *p* < 0.001 and *q* < 0.001 for each correlation). Vitamin B12 and folate intake were significantly correlated with their serum level (ρ = 0.234 and ρ = 0.325 respectively in men, ρ = 0.111 and ρ = 0.447 respectively in women, *p* < 0.001 and *q* < 0.001 for each correlation) and sodium intake was significantly correlated with its urinary level both in men and women (ρ = 0.183 in men, ρ = 0.185 in women, *p* < 0.001, and *q* < 0.001 for each correlation).

Some significant differences between correlations in men and women were found by Fisher's z-test (energy intake with pTEE, *p* = 0.017; vitamin B12 intake with its serum level, *p* = 0.034; folate intake with its serum level, *p* = 0.016) but these gender differences were no longer significant after the correction for multiple testing (*q* = 0.319, *q* = 0.538, *q* = 0.319 respectively).

The above mentioned correlations were evaluated also in each NU-AGE study center (Supplementary Table 3) and no significant differences were observed among countries (data not shown).

Considering that the bioavailability and adsorption of vitamin B12 depend on many factors and is particularly critical in elderly, the association between the intake of vitamin B12 and its serum level was evaluated also by a general linear model with backward elimination in the entire NU-AGE population, in men and in women (Table 4 and Supplementary Tables 4–6). The initial model exploited dietary intake of vitamin B12, age, alcohol consumption, use of proton pump inhibitors (PPI), SNAQ score and chewing difficulties as regressors. In the entire NU-AGE population, alcohol consumption is negatively associated (*p* = 0.004) while vitamin B12 intake was positively associated (*p* < 0.001) with serum level of vitamin B12. In men and in women, vitamin B12 intake was positively associated with serum level of vitamin B12 (*p* < 0.001 for both gender). In women, also SNAQ score is positively associated with serum level of vitamin B12 (*p* = 0.046).

**Table 4:** Association between the intake of Vitamin B12 and the serum level of vitamin B12 in the entire NU-AGE population, in men and women.

		**Vitamin B12 (serum)**	
	**Indipendent variables**	**β coefficient (95% C.I.)**	***p***
Entire population	Alcohol intake	−0.032 (−0.053 to −0.010)	0.004
	Vitamin B12 intake	0.063 (0.040 to 0.085)	<0.001
Men	Vitamin B12 intake	0.075 (0.045 to 0.103)	<0.001
Women	Vitamin B12 intake	0.054 (0.022 to 0.085)	0.001
	SNAQ score	0.291 (0.005 to 0.577)	0.046

*After the logarithmic transformation of the variables, the association between the intake of vitamin B12 and its serum level was evaluated by a general linear model with backward elimination adjusted for age, alcohol intake, use of PPI, SNAQ score and chewing difficulties*.

A general linear regression model was performed also to identify the variables associated with plasma level of homocysteine in the entire NU-AGE population and by gender (Table 5 and Supplementary Tables 7–9). The initial model exploited the dietary intake of vitamin B2, B6, B12, and folate, age, alcohol intake, use of PPI, SNAQ score, and chewing difficulties as regressors. In the entire NU-AGE population, age was positively associated (*p* = 0.004) while vitamin B12 and folate intake were negatively associated with plasma level of homocysteine (*p* = 0.001 and *p* = 0.004, respectively). In men, alcohol intake and the use of PPI were moderately positively associated (*p* = 0.021 and *p* = 0.054, respectively) while vitamin B6 and vitamin B12 were negatively associated (*p* = 0.004 and *p* = 0.003, respectively) with plasma level of homocysteine. In women, folate intake was negatively associated (*p* = 0.006) and vitamin B12 intake was slightly negatively associated with plasma level of homocysteine (*p* = 0.073).

**Table 5:** Predictors of plasma levels of homocysteine in the entire NU-AGE population, in men and women.

		**Homocysteine (plasma)**	
	**Indipendent variables**	**β coefficient (95% C.I.)**	**p**
Entire population	Age	0.005 (0.001–0.010)	0.025
	Vitamin B12 intake	−0.033 (−0.053 to −0.014)	0.001
	Folate intake	−0.069 (−0.115 to −0.022)	0.004
Men	Alcohol intake	0.027 (0.050 to 0.004)	0.021
	Use of PPI	0.075 (0.152 to 0.001)	0.054
	Vitamin B6 intake	−0.089 (−0.149 - −0.030)	0.004
	Vitamin B12 intake	−0.044 (-0.073 to −0.015)	0.003
Women	Folate intake	−0.083 (-0.142 to −0.024)	0.006
	Vitamin B12 intake	−0.024 (-0.051 to −0.002)	0.073

*After the logarithmic transformation of the variables, association between dietary intake of vitamin B2, B6, B12, and folate with plasma level of homocysteine was evaluated by a general linear model with backward elimination, adjusted for age, alcohol intake, use of PPI, SNAQ score and chewing difficulties*.

## Discussion

The assessment of dietary intake is a fundamental aspect to investigate the role of nutrition together with lifestyle, environmental, and genetic risk factors in preventing or predisposing to age-related diseases.

For the first time, the present study have analyzed the correlation/association between the daily intake of selected nutrients (estimated by a 7-day food records) and blood/urine biomarkers of intake on a unprecedented consistent population of healthy elderly subjects (*n* = 1,140) across five European centers. Dietary intake was assessed by means of 7-day food records (combined with a questionnaire for the mineral/vitamin supplements) according a standardized method designed for the participants of NU-AGE project (see section Materials and Method for details) at the baseline of the NU-AGE dietary intervention.

On the whole, the results of this study on the entire NU-AGE population indicated that dietary intakes calculated from 7-day food records are significantly correlated/associated with pTEE as well as with blood and urine biomarkers. However, some of the univariate correlation are weak.

In particular, daily energy intake is significantly correlated with pTEE. Nevertheless, the median energy intake is lower than pTEE in entire population and in both genders. This could mean that participants have underreported their food consumption in some cases or that they have undereating during the period of recording. Divergences between reported energy intakes and measured energy expenditures have been described in obese subjects (Schoeller et al., 1990). In this study, the total energy expenditure has been not measured but predicted by a formula taking into account basal metabolic rate and the level of physical activity. For the calculation of basal metabolic rate, we used the Mifflin-St. Jeor equation which has been demonstrated to be the most accurate method of estimating resting metabolic rate in postmenopausal women (Bonganha et al., 2016) and in healthy people of various body sizes (Frankenfield, 2013). However, the accuracy rate of the Mifflin St. Jeor equations is lower in obese than non-obese people and maintain a slight bias toward overestimation. Analysis by gender showed some important differences between men and women. The correlation between total energy intake and pTEE is significant only in men. Such discrepancy may be explained by a certain grade of underreporting of usual food consumption. In a group of lean women, the underreporting in the food records was entirely explained by undereating: subjects changed their diet decreasing their food consumption during the observation period (Goris and Westerterp, 1999). Another study have analyzed the behavior of group of non-obese women kept for a day in a metabolic facility with *ad libitum* food intake which was covertly measured. The next day, subjects wrote down what they ate and drank during the previous 24 h. Food items eaten during a meal were reported accurately, but the between-meal snack foods were underreported. The snacks provided were highly caloric and carbohydrate rich and there was a selective underreporting of carbohydrates (Poppitt et al., 1998). The underreporting in a group of obese men has been explained by undereating but also by under-recording: subjects selectively underreported fat intake and carbohydrate-rich foods while the underreporting of protein was disproportional and less frequent (Goris et al., 2000). Accordingly, in the present work, protein intake seems to be consistently estimated by 7-day food records. Indeed, our data showed quite robust correlations between a reliable index of recent dietary protein intake (ratio of 24 h urinary urea to creatinine excretion) (Tay et al., 2015; Di Girolamo et al., 2017) and total protein intake, animal protein intake and protein intake/BW both in men and in women reinforcing the validity of 7-day food records for the estimation of protein foods consumption.

Folate and vitamin B12 are essential nutrients, and their deficiencies represent public health problems worldwide, affecting all age groups and leading to complications such as anemia, birth defects, and neurological disorders (de Benoist, 2008; Barnabé et al., 2015). These vitamins are key players for the catalysis of methyl group transfer in methylation reactions, essential for life maintenance (Kung et al., 2012). Serum concentrations of these vitamins are considered biomarkers of the status of this micronutrients in the organism, indeed reflecting long-term dietary composition but they cannot be directly translated to their absolute intakes because their blood levels are influenced by numerous physiological and environmental factors in addition to diet (Yuan et al., 2017). Despite these limitations, data from the present study showed a moderate correlation between folate intake and its serum level (ρ = 0.363). This correlation was significant also dividing NU-AGE subjects by gender (ρ = 0.325 for men and ρ = 0.447 for women). A qualitative review considering 17 validation studies showed that the strength of correlations between folate intake assessed by different methods (food frequency questionnaire, 24 h dietary recall, food records) and the blood folate concentration varied from weak to moderate (*r* = 0.05–0.54) (Park et al., 2013). Even if blood folate level may depend on a few variables (genetic polymorphisms, specific drugs, alcoholism, conditions associated with increased cell turnover as well as the bioavailability of folate from food) (Barnabé et al., 2015; Saini et al., 2016), jejunum folate resorption by enterocytes receptors via endocytosis (Wibowo et al., 2013) is of no clinical concern unless the presence of viral infection (Xiao et al., 2012). On the other hand, the bioavailability and adsorption of vitamin B12 is more critical particularly in elderly because the frequency of gastritis or inflammation of the gastric mucosa increases with age and results in a reduction, or in some cases, complete loss of the acid required to cleave vitamin B12 from food protein (O'Leary and Samman, 2010). Thus, aging and frailty may cause intrinsic vitamin B12 deficiencies (which can occur independently of nutritional intake) and the blood level of vitamin B12 is considered a shortcoming concentration marker of vitamin 12 intake, particularly in elderly. However, our study showed a weak but significant correlation between vitamin B12 intake and its serum level in entire NU-AGE population as well as in men and in women. Even a general linear model considering alcohol intake, age and use of proton pump inhibitor drugs showed a significant association between vitamin B12 intake and its serum level in the entire NU-AGE population, in men and women, suggesting a good performance of NU-AGE food records in estimating its intake and a preserved vitamin B12 absorption capacity of NU-AGE elderly participants.

Folate and vitamin B12 are necessary for the methylation of homocysteine to methionine (Edney et al., 2015; Kennedy, 2016). Vitamin B6 is an essential cofactor for the homocysteine trans-sulfuration pathway that can catabolize effectively the potentially toxic excess of homocysteine not required for methyl transfer (Pandey and Pradhan, 2006). Vitamin B2 may also influence homocysteine pathway indirectly via its role as cofactor for methylenetetrahydrofolate reductase (MTHFR), enzyme that catalyzes the conversion of 5,10-methylenetetrahydrofolate to 5-methyltetrahydrofolate, a co-substrate for homocysteine remethylation to methionine (Anderson et al., 2012). Different clinical trials exploited the role of supplements with a combination of folate and vitamins B6 and/or B12 to normalize homocysteine level (Smith and Refsum, 2016). Indeed, low intake of folate and vitamin B12 are associated with high homocysteine levels considered a risk factor for cardiovascular disease, dementia and depression (Tiemeier et al., 2002; Moretti et al., 2004; Zakai et al., 2007; Kim et al., 2008; Smith et al., 2018). Our results confirmed a negative association between the intake of vitamin B12 and folate with homocysteine level in plasma but some interesting differences between genders are found. In men, vitamin B6 and vitamin B12 intake are negatively associated while alcohol intake and the use of PPI are positively associated with plasma level of homocysteine. In women, folate and vitamin B12 intake are negatively associated with plasma level of homocysteine. This gender differences could explain some contrasting results obtained by many intervention studies administering just folate and vitamin B12 on the basis that increasing the levels of these vitamins will reliably reduce homocysteine levels and thus the risk of cardiocerebrovascular events and all-cause mortality (Mei et al., 2010; Kennedy, 2016). Moreover, our results may suggest the importance of gender specific intervention/recommendation to control blood homocysteine level in elderly, i.e., reducing alcohol intake and to improving assumption/adsorption of vitamin B6 and B12 in men and increasing the intake of folate and vitamin B12 in women. The median intakes of vitamin B12 and alcohol satisfied the nutrient requirements of the NU-AGE diet while the intake of folate at the baseline had to be augmented (Berendsen et al., 2014). Accordingly, the consumption of folate rich foods (fruits, vegetables, legumes, and nuts) has been strongly encouraged during the NU-AGE dietary intervention and it resulted significantly increased in the NU-AGE diet group.

The use of recovery biomarkers of absolute intake (sodium and potassium concentration in 24-h urine) is the recommended method for assessing daily sodium and potassium intake. Indeed, 90% of sodium is excreted through urine and measurements on urine do not rely on self-report information. Our data show a significant correlation between sodium intake and its urinary level which maintains its significance also dividing subjects in men and women. This result is particularly interesting considering that many dietary methods assessment tend to underestimate sodium intakes due to the difficulties on quantifying salt present in the processed foods and added during cooking and/or at the table (James et al., 1987; Mattes and Donnelly, 1991; Magriplis et al., 2011; Lerchl et al., 2015). The coefficient of correlation between the estimation of intake of sodium and its 24 h urinary measures found in the present study (0.297 for the entire study population) is lower but in line with the coefficient of correlation (0.36) found in the study of Day et al. (2001). Also the assessment of potassium intake by 24 h urine measurement is important because potassium chloride may be used as a salt substitute to replace sodium chloride, although potassium excretion may be more variable (77–90%) (John et al., 2016). In the present study, potassium intake is weakly correlated to urinary potassium level considering the entire NU-AGE population and this correlation is no longer significant dividing subjects by gender. On the contrary, a previous study on 179 members of the EPIC-Norfolk cohort aimed to the comparison of the performance of a 7-day records with a food frequency questionnaire using urinary markers, found a robust correlation between potassium intake resulting from the 7-day food records and 24 h urinary potassium excretion (Day et al., 2001).

Despite many strengths, i.e., the consistent number of subjects recruited in five different European countries, the use of a 7-day food records with a standardized protocol among the countries, the reliability of nutrients biomarkers, our study has some limitations. Firstly, the use of country specific food composition databases and nutrient calculation systems could have resulted in different intakes among the countries. Secondly, the energy expenditure has been estimated and not actually measured. Finally, participants were apparently healthy, with no noteworthy problem of appetite, chewing and depression and, being volunteers, interested in nutrition, and health topics, which limits the generalizability of our findings to the general elderly population.

The 7-day food record is a validate and interesting method to collect data about the foods and beverages consumed over a limited period of time. Even if a good validity and precision has been reported for the method if adequate procedures are employed and a sufficient number of days are considered, this method is affected by error and presents some limitations due mainly to the high burden posed on respondents (i.e., the possibility that some subjects to modify food behavior in order to simplify the registration of food intake, the tendency to report food consumption close to those socially desirable and the difficulties for some individuals who do not cook regularly and are not familiar with weighing foods in reporting their intakes) (Ortega et al., 2015). Moreover, the concentration of blood and urinary nutrient biomarkers depend on several factors such as bioavailability, therapies, age, diseases, gut microbiota composition, etc. and reflect their absorption and metabolism after consumption and not exactly the subject's absolute dietary intake. However, the present study found significant (even if weak in some cases) correlation/association between daily reported intake of selected nutrients (protein, vitamin B12, folate, sodium) and their blood/urinary markers possibly confirming that the 7-day food records (plus a supplementation questionnaire) may provide reliable data to evaluate current food intake and to monitor the progress of dietary intervention in healthy elderly subjects. In particular, during NU-AGE project, 7-day food records has been effectively employed by the dieticians/nutritionists to assess the dietary intakes at the baseline and to guide NU-AGE participants through the shift of their dietary habits according NU-AGE dietary recommendations.

## Author contributions

RO, AS, and CF contributed to the conception and design of the current work. RO, GG, and EG contributed to data analyses and data interpretation. RO drafted the manuscript. CF conceived, designed, initiated and directed the NU-AGE project. AS co-ordinated NU-AGE data collection across centers. AB and LdG designed the NU-AGE dietary intervention/diet. RO, CL, AB, OJ, ES, BP, NL, and EC followed the dietary intervention. AB, OJ, ES, BP, AJ, RG, NM, EF, SF-T, and MC substantially contributed to the data collection by acquiring or processing data. All authors contributed to interpretation of data, critically revised, and approved the final version of this manuscript.

### Conflict of interest statement

The authors declare that the research was conducted in the absence of any commercial or financial relationships that could be construed as a potential conflict of interest.
