# Supplementary material for: Cross-Sectional Analysis of the Correlation Between Daily Nutrient Intake Assessed by 7-Day Food Records and Biomarkers of Dietary Intake Among Participants of the NU-AGE Study
Source: Front Physiol. 2018 Oct 1;9:1359. doi: 10.3389/fphys.2018.01359 (PMC6174234; doi:10.3389/fphys.2018.01359)
Supplement: Supplementary file 1 [file Data_Sheet_1.pdf]

The structured format of 7-day food record is reported below for the first day. The model is repeated for 7 days.

Name and surname: ____ **CODE:** ____________________________

DATE: ___________________________________ **DAY OF THE WEEK:** _____________________________________

DAY 1
Before breakfast:

Time	Place	DESCRIPTION OF FOODS/DRINKS AND PREPARATION	QUANTITY (as precise as possible)

Breakfast:

Time	Place	DESCRIPTION OF FOODS/DRINKS AND PREPARATION	QUANTITY (as precise as possible)

Name and surname: ________________________________ CODE: ______________________

DATE: ____________________________ DAY OF THE WEEK: ______________________________

DAY 1

During the morning:

Time	Place	DESCRIPTION OF FOODS/DRINKS AND PREPARATION	QUANTITY (as precise as possible)

Lunch:

Time	Place	DESCRIPTION OF FOODS/DRINKS AND PREPARATION	QUANTITY (as precise as possible)

Name and surname: ____ CODE: ________________________________

DATE: ____ DAY OF THE WEEK: ____

DAY 1

During the afternoon:

Time	Place	DESCRIPTION OF FOODS/DRINKS AND PREPARATION	QUANTITY (as precise as possible)

Dinner:

Time	Place	DESCRIPTION OF FOODS/DRINKS AND PREPARATION	QUANTITY (as precise as possible)

Name and surname: ______________________________ CODE: ____________________

DATE: __________________________ DAY OF THE WEEK: ____________________________

DAY 1

After dinner:

Time	Place	DESCRIPTION OF FOODS/DRINKS AND PREPARATION	QUANTITY (as precise as possible)

During the night:

Time	Place	DESCRIPTION OF FOODS/DRINKS AND PREPARATION	QUANTITY (as precise as possible)

Note (es: recipes)

____
____
____
____
____
____
