# Supplementary material for: Cross-Sectional Analysis of the Correlation Between Daily Nutrient Intake Assessed by 7-Day Food Records and Biomarkers of Dietary Intake Among Participants of the NU-AGE Study
Source: Front Physiol. 2018 Oct 1;9:1359. doi: 10.3389/fphys.2018.01359 (PMC6174234; doi:10.3389/fphys.2018.01359)
Supplement: Supplementary file 2 [file Table_1.pdf]

Supplementary table 1. Per-nutrient percentage variability among NU-AGE study centers obtained by the analysis of 16 foods by means of food composition databases of each country.

	**Per-nutrient variability among centers (%)**
**Energy**	5.8
**Carbohydrates**	14.3
**Lipid**	19.4
**Protein**	5.9
**Vitamin B2**	22.8
**Vitamin B6**	16.6
**Vitamin B12**	3.0
**Folate**	22.2
**Potassium**	8.4
**Sodium**	26.1
