# Supplementary material for: Cross-Sectional Analysis of the Correlation Between Daily Nutrient Intake Assessed by 7-Day Food Records and Biomarkers of Dietary Intake Among Participants of the NU-AGE Study
Source: Front Physiol. 2018 Oct 1;9:1359. doi: 10.3389/fphys.2018.01359 (PMC6174234; doi:10.3389/fphys.2018.01359)
Supplement: Supplementary file 3 [file Table_2.pdf]

Supplementary table 2. Spearman rank correlation between daily dietary intakes, pTEE, blood and urine biomarkers not excluding and excluding mis-reporting subjects (120 under-reporters, 10.5% of the participants and 49 over-reporters, 4.3% of the participants) in the entire NU-AGE population and by gender.

	Not excluding mis-reporting		Excluding mis-reporting		Not excluding mis-reporting (men)		Excluding mis-reporting (men)		Not excluding mis-reporting (women)		Excluding mis-reporting (women)	
	ρ (rho)	p	ρ (rho)	p	ρ (rho)	p	ρ (rho)	p	ρ (rho)	p	ρ (rho)	p
**Energy intake*pTEE**	0.459	<0.001	0.627	<0.001	0.206	<0.001	0.313	<0.001	0.066	0.099	0.076	0.101
**Total protein intake*Urea:creatinine excretion[a]**	0.101	<0.001	0.104	<0.001	0.413	<0.001	0.403	<0.001	0.354	<0.001	0.351	<0.001
**Animal protein intake*Urea:creatinine excretion[#a]**	0.264	<0.001	0.249	<0.001	0.412	<0.001	0.417	<0.001	0.401	<0.001	0.369	<0.001
**Protein intake/BW (g/kg BW)*Urea:creatinine excretion[a]**	0.352	<0.001	0.340	<0.001	0.423	<0.001	0.425	<0.001	0.403	<0.001	0.408	<0.001
**Vitamin B12 intake*Vitamin B12 (serum)**	0.151	<0.001	0.152	<0.001	0.234	<0.001	0.218	<0.001	0.111	<0.001	0.135	0.002
**Folate intake*Folate (serum)**	0.363	<0.001	0.364	<0.001	0.325	<0.001	0.332	<0.001	0.447	<0.001	0.460	<0.001
**Potassium intake*Potassium (urine)[a]**	0.148	<0.001	0.127	<0.001	0.065	0.176	0.039	0.429	0.062	0.154	0.039	0.363
**Sodium intake*Sodium (urine)[a]**	0.297	<0.001	0.350	<0.001	0.183	<0.001	0.227	<0.001	0.185	<0.001	0.232	<0.001
