# Supplementary material for: Cross-Sectional Analysis of the Correlation Between Daily Nutrient Intake Assessed by 7-Day Food Records and Biomarkers of Dietary Intake Among Participants of the NU-AGE Study
Source: Front Physiol. 2018 Oct 1;9:1359. doi: 10.3389/fphys.2018.01359 (PMC6174234; doi:10.3389/fphys.2018.01359)
Supplement: Supplementary file 4 [file Table_3.pdf]

Supplementary table 3. Spearman rank correlation between daily dietary intakes, pTEE, blood and urine biomarkers in each NU-AGE study center (country).

	Italy (n=242)			UK (n=248)			The Netherlands(n=241)			Poland (n=220)			France (n=189)		
	**ρ**	**p**	**q**	**ρ**	**p**	**q**	**ρ**	**p**	**q**	**ρ**	**p**	**q**	**ρ**	**p**	**q**
**Energy intake*pTEE**	0.452	<0.001	<0.001	0.484	<0.001	<0.001	0.322	<0.001	<0.001	0.395	<0.001	<0.001	0.615	<0.001	<0.001
**Total protein intake*Urea:creatinine excretion[a]**	0.113	0.090	0.998	0.014	0.847	0.998	0.127	0.084	0.998	0.094	0.203	0.998	0.039	0.612	0.998
**Animal protein intake*Urea:creatinine excretion[a]**	0.011	0.864	0.998	0.084	0.233	0.998	0.128	0.083	0.998	0.154	0.037	0.998	n.a.	n.a.	n.a.
**Protein intake/BW (g/kg BW)*Urea:creatinine excretion[a]**	0.194	0.003	0.111	0.375	<0.001	<0.001	0.388	<0.001	<0.001	0.313	<0.001	<0.001	0.277	<0.001	<0.001
**Vitamin B12 intake*Vitamin B12 (serum)**	0.171	0.008	0.272	0.138	0.030	0.900	0.199	0.002	0.080	0.030	0.654	0.998	-0.040	0.582	0.998
**Folate intake*Folate (serum)**	0.387	<0.001	<0.001	0.488	<0.001	<0.001	0.397	<0.001	<0.001	0.198	0.003	0.111	0.331	<0.001	<0.001
**Potassium intake*Potassium (urine)[a]**	0.134	0.044	0.998	0.053	0.458	0.998	0.085	0.247	0.998	0.224	0.002	0.080	0.213	0.005	0.175
**Sodium intake*Sodium (urine)[a]**	0.332	<0.001	<0.001	0.299	<0.001	<0.001	0.296	<0.001	<0.001	0.392	<0.001	<0.001	0.277	<0.001	<0.001

ρ (rho) is the Spearman rank correlation coefficient and p the significance of Spearman rank correlation. q-values derive from Benjamini-Hockberg correction of p-values for multiple testing. Animal protein intake was not assessed on French participants. [a]169 subjects (16 for Italy, 46 for UK, 56 for The Netherlands and 15 for France) were excluded because outside of the expected range of creatinine excretion in relation to body weight.
