# Supplementary material for: Cross-Sectional Analysis of the Correlation Between Daily Nutrient Intake Assessed by 7-Day Food Records and Biomarkers of Dietary Intake Among Participants of the NU-AGE Study
Source: Front Physiol. 2018 Oct 1;9:1359. doi: 10.3389/fphys.2018.01359 (PMC6174234; doi:10.3389/fphys.2018.01359)
Supplement: Supplementary file 5 [file Table_4.pdf]

Supplementary table 4. Predictors of serum level of Vitamin B12 in the entire NU-AGE population.

		Vitamin B12 (serum)	
	**Indipendent variables**	**β coefficient (95% C.I.)**	**p**
**Model 1**	Age	-0.003 (-0.009 - 0.003)	0.286
	Vitamin B12 intake	0.061 (0.039 - 0.083)	<0.001
	Alcohol intake	-0.036 (-0.057 - -0.014)	0.001
	Use of PPI	0.001 (-0.057 - 0.060)	0.970
	SNAQ score	0.142 (-0.070 - 0.354)	0.190
	Chewing difficulties	0.027 (-0.055 - 0.108)	0.520
**Model 2**	Age	-0.003 (-0.009 - 0.003)	0.284
	Vitamin B12 intake	0.061 (0.039 - 0.083)	<0.001
	Alcohol intake	-0.036 (-0.057 - -0.014)	0.001
	SNAQ score	0.142 (-0.069 - 0.354)	0.187
	Chewing difficulties	0.027 (-0.054 - 0.108)	0.520
**Model 3**	Age	-0.003 (-0.009 - 0.002)	0.207
	Vitamin B12 intake	0.062 (0.039 - 0.084)	<0.001
	Alcohol intake	-0.034 (-0.056 - -0.013)	0.002
	SNAQ score	0.149 (-0.063 - 0.360)	0.168
**Model 4**	Vitamin B12 intake	0.063 (0.041 - 0.085)	<0.001
	Alcohol intake	-0.033 (-0.055 - -0.012)	0.002
	SNAQ score	0.155 (-0.056 – 0.366)	0.149
**Model 5**	Vitamin B12 intake	0.063 (0.040 - 0.085)	<0.001
	Alcohol intake	-0.032 (-0.053- -0.010)	0.004
