# Supplementary material for: Cross-Sectional Analysis of the Correlation Between Daily Nutrient Intake Assessed by 7-Day Food Records and Biomarkers of Dietary Intake Among Participants of the NU-AGE Study
Source: Front Physiol. 2018 Oct 1;9:1359. doi: 10.3389/fphys.2018.01359 (PMC6174234; doi:10.3389/fphys.2018.01359)
Supplement: Supplementary file 6 [file Table_5.pdf]

Supplementary table 5. Predictors of serum level of vitamin B12 in men.

		**Vitamin B12 (serum)**	
	**Indipendent variables**	**β coefficient (95% C.I.)**	**p**
**Model 1**	Age	0.000 (-0.008 - 0.007)	0.921
	Vitamin B12 intake	0.076 (0.046 - 0.105)	<0.001
	Alcohol intake	-0.018 (-0.043 - 0.008)	0.180
	Use of PPI	0.045 (-0.041 - 0.130)	0.304
	SNAQ score	-0.064 (-0.373 - 0.245)	0.685
	Chewing difficulties	0.076 (-0.056 - 0.208)	0.257
**Model 2**	Vitamin B12 intake	0.076 (0.046 - 0.105)	<0.001
	Alcohol intake	-0.018 (-0.043 - 0.008)	0.181
	Use of PPI	0.045 (-0.040 - 0.130)	0.297
	SNAQ score	-0.063 (-0.371 - 0.245)	0.689
	Chewing difficulties	0.076 (-0.056 - 0.207)	0.258
**Model 3**	Vitamin B12 intake	0.076 (0.047 - 0.106)	<0.001
	Alcohol intake	-0.016 (-0.041 - 0.010)	0.219
	Use of PPI	0.044 (-0.040 - 0.129)	0.305
	Chewing difficulties	0.074 (-0.057 - 0.204)	0.269
**Model 4**	Vitamin B12 intake	0.077 (0.048 - 0.106)	<0.001
	Alcohol intake	-0.016 (-0.041 - 0.009)	0.214
	Chewing difficulties	0.074 (-0.056 - 0.205)	0.263
**Model 5**	Vitamin B12 intake	0.077 (0.048 - 0.106)	<0.001
	Alcohol intake	-0.016 (-0.041 - 0.010)	0.222
**Model 6**	Vitamin B12 intake	0.074 (0.045 - 0.103)	<0.001
