# Supplementary material for: Cross-Sectional Analysis of the Correlation Between Daily Nutrient Intake Assessed by 7-Day Food Records and Biomarkers of Dietary Intake Among Participants of the NU-AGE Study
Source: Front Physiol. 2018 Oct 1;9:1359. doi: 10.3389/fphys.2018.01359 (PMC6174234; doi:10.3389/fphys.2018.01359)
Supplement: Supplementary file 7 [file Table_6.pdf]

Supplementary table 6. Predictors of serum level of vitamin B12 in women.

		Vitamin B12 (serum)	
	**Indipendent variables**	**β coefficient (95% C.I.)**	**p**
**Model 1**	Age	-0.004 (-0.012 - 0.004)	0.304
	Vitamin B12 intake	0.052 (0.020 - 0.084)	0.002
	Alcohol intake	-0.027 (-0.068 - 0.014)	0.190
	Use of PPI	-0.023 (-0.101 - 0.056)	0.573
	SNAQ score	0.311 (0.022 - 0.599)	0.035
	Chewing difficulties	0.031 (-0.072 - 0.135)	0.553
**Model 2**	Age	-0.004 (-0.012 - 0.004)	0.309
	Vitamin B12 intake	0.052 (0.020 - 0.084)	0.002
	Alcohol intake	-0.028 (-0.069 - 0.013)	0.179
	SNAQ score	0.303 (0.016 - 0.591)	0.039
	Chewing difficulties	0.031 (-0.072 - 0.135)	0.552
**Model 3**	Age	-0.004 (-0.012 - 0.004)	0.293
	Vitamin B12 intake	0.052 (0.020 - 0.084)	0.001
	Alcohol intake	-0.028 (-0.068 - 0.013)	0.182
	SNAQ score	0.300 (0.016 - 0.587)	0.040
**Model 4**	Vitamin B12 intake	0.054 (0.022 - 0.086)	0.001
	Alcohol intake	-0.025 (-0.066 - 0.015)	0.219
	SNAQ score	0.305 (0.018 - 0.592)	0.037
**Model 5**	Vitamin B12 intake	0.054 (0.022 - 0.085)	0.001
	SNAQ score	0.291 (0.005 - 0.577)	0.046
