# Supplementary material for: Cross-Sectional Analysis of the Correlation Between Daily Nutrient Intake Assessed by 7-Day Food Records and Biomarkers of Dietary Intake Among Participants of the NU-AGE Study
Source: Front Physiol. 2018 Oct 1;9:1359. doi: 10.3389/fphys.2018.01359 (PMC6174234; doi:10.3389/fphys.2018.01359)
Supplement: Supplementary file 8 [file Table_7.pdf]

Supplementary table 7. Predictors of plasma levels of homocysteine in the entire NU-AGE population.

		**Homocysteine (plasma)**	
	**Indipendent variables**	**β coefficient (95% C.I.)**	**p**
**Model 1**	Age	0.005 (0.001 - 0.010)	0.026
	Vitamin B12 intake	-0.030 (-0.051 - -0.010)	0.004
	Folate intake	-0.039 (-0.099 - 0.020)	0.196
	Vitamin B2 intake	-0.015 (-0.069 - 0.038)	0.576
	Vitamin B6 intake	-0.023 (-0.083 - 0.036)	0.438
	Alcohol intake	0.004 (-0.014 - 0.023)	0.647
	Use of PPI	0.000 (-0.050 - 0.050)	0.989
	SNAQ score	-0.028 (-0.213 - 0.156)	0.763
	Chewing difficulties	0.044 (-0.026 - 0.113)	0.217
**Model 2**	Age	0.005 (0.001 - 0.010)	0.024
	Vitamin B12 intake	-0.030 (-0.051 - -0.010)	0.004
	Folate intake	-0.039 (-0.099 - 0.020)	0.196
	Vitamin B2 intake	-0.015 (-0.069 - 0.038)	0.577
	Vitamin B6 intake	-0.023 (-0.082 - 0.036)	0.437
	Alcohol intake	0.004 (-0.014 - 0.023)	0.635
	SNAQ score	-0.028 (-0.212 - 0.156)	0.767
	Chewing difficulties	0.044 (-0.025 - 0.113)	0.213
**Model 3**	Age	0.005 (0.001 - 0.010)	0.023
	Vitamin B12 intake	-0.030 (-0.051 - -0.009)	0.004
	Folate intake	-0.040 (-0.099 - 0.019)	0.179
	Vitamin B2 intake	-0.015 (-0.068 - 0.039)	0.592
	Vitamin B6 intake	-0.024 (-0.083 - 0.035)	0.418
	Alcohol intake	0.004 (-0.014 - 0.023)	0.639
	Chewing difficulties	0.044 (-0.025 - 0.113)	0.215
**Model 4**	Age	0.005 (0.001 - 0.010)	0.025
	Vitamin B12 intake	-0.030 (-0.050 - -0.009)	0.005
	Folate intake	-0.042 (-0.101 - 0.017)	0.161
	Vitamin B2 intake	-0.014 (-0.067 - 0.039)	0.600
	Vitamin B6 intake	-0.024 (-0.083 - 0.035)	0.425
	Chewing difficulties	0.044 (-0.025 - 0.113)	0.208
**Model 5**	Age	0.005 (0.001 - 0.010)	0.026
	Vitamin B12 intake	-0.031 (-0.051 - -0.010)	0.003
	Folate intake	-0.045 (-0.103 - 0.012)	0.123
	Vitamin B6 intake	-0.033 (-0.081 - 0.014)	0.172
	Chewing difficulties	0.045 (-0.024 - 0.114)	0.201
**Model 6**	Age	0.005 (0.001 - 0.010)	0.028
	Vitamin B12 intake	-0.030 (-0.050 - -0.009)	0.004
	Folate intake	-0.044 (-0.101 - 0.014)	0.136
	Vitamin B6 intake	-0.036 (-0.083 - 0.012)	0.141
**Model 7**	Age	0.005 (0.001 - 0.010)	0.026
	Vitamin B12 intake	-0.033 (-0.053 - -0.014)	0.001
	Folate intake	-0.069 (-0.115 - 0.022)	0.004
