# Supplementary material for: Cross-Sectional Analysis of the Correlation Between Daily Nutrient Intake Assessed by 7-Day Food Records and Biomarkers of Dietary Intake Among Participants of the NU-AGE Study
Source: Front Physiol. 2018 Oct 1;9:1359. doi: 10.3389/fphys.2018.01359 (PMC6174234; doi:10.3389/fphys.2018.01359)
Supplement: Supplementary file 9 [file Table_8.pdf]

Supplementary table 8. Predictors of plasma levels of homocysteine in women.

		Homocysteine (plasma)	
	**Indipendent variables**	**β coefficient (95% C.I.)**	**p**
**Model 1**	Age	0.004 (-0.003 - 0.010)	0.257
	Vitamin B12 intake	-0.021 (-0.049 - 0.006)	0.132
	Folate intake	-0.068 (-0.144 - 0.009)	0.084
	Vitamin B2 intake	-0.006 (-0.074 - 0.062)	0.867
	Vitamin B6 intake	-0.010 (-0.091 - 0.071)	0.806
	Alcohol intake	-0.012 (-0.044 - 0.020)	0.479
	Use of PPI	0.031 (-0.031 - 0.092)	0.329
	SNAQ score	-0.041 (-0.267 - 0.186)	0.724
	Chewing difficulties	0.050 (-0.0.31 - 0.131)	0.225
**Model 2**	Age	0.004 (-0.002 - 0.010)	0.230
	Vitamin B12 intake	-0.022 (-0.049 - 0.005)	0.115
	Folate intake	-0.069 (-0.145 - 0.007)	0.077
	Vitamin B6 intake	-0.015 (-0.076 - 0.047)	0.637
	Alcohol intake	-0.012 (-0.044 - 0.020)	0.451
	Use of PPI	0.032 (-0.029 - 0.093)	0.300
	SNAQ score	-0.041 (-0.266 - 0.184)	0.720
	Chewing difficulties	0.049 (-0.0.31 - 0.129)	0.227
**Model 3**	Age	0.004 (-0.002 - 0.010)	0.226
	Vitamin B12 intake	-0.022 (-0.049 - 0.006)	0.119
	Folate intake	-0.070 (-0.145 - 0.006)	0.072
	Vitamin B6 intake	-0.015 (-0.076 - 0.046)	0.622
	Alcohol intake	-0.013 (-0.044 - 0.019)	0.434
	Use of PPI	0.031 (-0.029 - 0.092)	0.313
	Chewing difficulties	0.050 (-0.030 - 0.130)	0.223
**Model 4**	Age	0.004 (-0.002 - 0.010)	0.229
	Vitamin B12 intake	-0.023 (-0.050 - 0.004)	0.089
	Folate intake	-0.082 (-0.141 - -0.022)	0.007
	Alcohol intake	-0.013 (-0.045 - 0.018)	0.399
	Use of PPI	0.031 (-0.029 - 0.092)	0.313
	Chewing difficulties	0.051 (-0.029 - 0.131)	0.211
**Model 5**	Age	0.004 (-0.002 - 0.010)	0.193
	Vitamin B12 intake	-0.024 (-0.050 - 0.003)	0.082
	Folate intake	-0.080 (-0.139 - -0.021)	0.008
	Use of PPI	0.030 (-0.030 - 0.090)	0.332
	Chewing difficulties	0.051 (-0.029 - 0.130)	0.214
**Model 6**	Age	0.004 (-0.002 - 0.010)	0.202
	Vitamin B12 intake	-0.024 (-0.051 - 0.003)	0.080
	Folate intake	-0.080 (-0.139 - -0.021)	0.008
	Chewing difficulties	0.050 (-0.029 - 0.130)	0.216
**Model 7**	Age	0.004 (-0.002 - 0.010)	0.226
	Vitamin B12 intake	-0.023 (-0.050 - 0.004)	0.092
	Folate intake	-0.083 (-0.141 - -0.024)	0.006
**Model 8**	Vitamin B12 intake	-0.024 (-0.051 - 0.002)	0.072
	Folate intake	-0.084 (-0.143 - -0.025)	0.005
