# Supplementary material for: Cross-Sectional Analysis of the Correlation Between Daily Nutrient Intake Assessed by 7-Day Food Records and Biomarkers of Dietary Intake Among Participants of the NU-AGE Study
Source: Front Physiol. 2018 Oct 1;9:1359. doi: 10.3389/fphys.2018.01359 (PMC6174234; doi:10.3389/fphys.2018.01359)
Supplement: Supplementary file 10 [file Table_9.pdf]

Supplementary table 9. Predictors of plasma levels of homocysteine in men.

		**Homocysteine (plasma)**	
	**Indipendent variables**	**β coefficient (95% C.I.)**	**p**
**Model 1**	Age	0.004 (-0.003 - 0.011)	0.232
	Vitamin B12 intake	-0.042 (-0.071 - -0.012)	0.006
	Folate intake	-0.005 (-0.095 - 0.085)	0.910
	Vitamin B2 intake	-0.034 (-0.117 - 0.049)	0.426
	Vitamin B6 intake	-0.064 (-0.147 - 0.018)	0.127
	Alcohol intake	0.027 (0.050 - 0.003)	0.028
	Use of PPI	-0.067 (-0.145 - 0.011)	0.094
	SNAQ score	-0.037 (-0.329 - 0.256)	0.805
	Chewing difficulties	-0.040 (-0.161 - 0.082)	0.522
**Model 2**	Age	0.004 (-0.003 - 0.011)	0.232
	Vitamin B12 intake	-0.042 (-0.071 - -0.012)	0.006
	Vitamin B2 intake	-0.035 (-0.114 - 0.043)	0.379
	Vitamin B6 intake	-0.065 (-0.145 - 0.014)	0.108
	Alcohol intake	0.027 (0.050 - 0.003)	0.028
	Use of PPI	-0.067 (-0.145 - 0.011)	0.092
	SNAQ score	-0.040 (-0.327 -0.247)	0.785
	Chewing difficulties	-0.040 (-0.161 - 0.080)	0.512
**Model 3**	Age	0.004 (-0.003 - 0.011)	0.225
	Vitamin B12 intake	-0.041 (-0.071 - -0.012)	0.006
	Vitamin B2 intake	-0.035 (-0.114 - 0.044)	0.382
	Vitamin B6 intake	-0.067 (-0.146 - 0.012)	0.095
	Alcohol intake	0.026 (0.050 - 0.003)	0.028
	Use of PPI	-0.068 (-0.146 - 0.010)	0.090
	Chewing difficulties	0.042 (-0.162 - 0.077)	0.487
**Model 4**	Age	0.004 (-0.003 - 0.010)	0.280
	Vitamin B12 intake	-0.041 (-0.071 - -0.012)	0.006
	Vitamin B2 intake	-0.035 (-0.114 - 0.043)	0.378
	Vitamin B6 intake	-0.066 (-0.145 - 0.012)	0.099
	Alcohol intake	0.026 (0.049 - 0.002)	0.032
	Use of PPI	-0.069 (-0.146 - 0.009)	0.085
**Model 5**	Age	0.003 (-0.003 - 0.010)	0.308
	Vitamin B12 intake	-0.044 (-0.072 - -0.015)	0.003
	Vitamin B6 intake	-0.089 (-0.149 - -0.029)	0.004
	Alcohol intake	0.026 (0.049 - 0.002)	0.032
	Use of PPI	-0.070 (-0.147 - 0.008)	0.080
**Model 6**	Vitamin B12 intake	-0.044 (-0.073 - -0.015)	0.003
	Vitamin B6 intake	-0.090 (-0.150 - -0.030)	0.003
	Alcohol intake	0.026 (0.050 - 0.003)	0.026
	Use of PPI	-0.073 (-0.151 - 0.004)	0.064
